# Low Prevalence of *TP53* Mutations and *MDM2* Amplifications in Pediatric Rhabdomyosarcoma

**DOI:** 10.1155/2012/492086

**Published:** 2012-03-07

**Authors:** Simona Ognjanovic, Ghyslaine Martel, Carlos Manivel, Magali Olivier, Erica Langer, Pierre Hainaut

**Affiliations:** ^1^Division of Pediatric Epidemiology and Clinical Research, University of Minnesota, 420 Delaware Street SE, MMC 422, Minneapolis, MN 55455, USA; ^2^Masonic Cancer Center, University of Minnesota, 425 E. River Road, 554 MCRB, Minneapolis, MN 55455, USA; ^3^Department of Pediatrics, University of Minnesota MMC 715, 420 Delaware Street SE, Minneapolis MN 55455, USA; ^4^Section of Molecular Carcinogenesis, International Agency for Research on Cancer, 150 Cours Albert Thomas, 69372 Lyon Cedex 08, France; ^5^Department of Laboratory Medicine and Pathology, University of Minnesota, Minneapolis, MN 55455, USA

## Abstract

The tumor suppressor gene *TP53* is the most commonly mutated gene in human cancer. The reported prevalence of mutations in rhabdomyosarcoma (RMS) varies widely, with recent larger studies suggesting that *TP53* mutations in pediatric RMS may be extremely rare. Overexpression of MDM2 also attenuates p53 function. We have performed *TP53* mutation/*MDM2* amplification analyses in the largest series analyzed thus far, including DNA isolated from 37 alveolar and 38 embryonal RMS tumor samples obtained from the Cooperative Human Tissue Network (CHTN). Available samples were frozen tumor tissues (*N* = 48) and histopathology slides. *TP53* mutations in exons 4–9 were analyzed by direct sequencing in all samples, and *MDM2* amplification analysis was performed by differential PCR on a subset of 22 samples. We found only one sample (1/75, 1.3%) carrying a *TP53* mutation at codon 259 (p.D259Y) and no *MDM2* amplification. Two SNPs in the *TP53* pathway, associated with accelerated tumor onset in germline *TP53* mutation carriers, (*TP53* SNP72 (rs no. 1042522) and MDM2 SNP309 (rs no. 2279744)), were not found to confer earlier tumor onset. In conclusion, we confirm the extremely low prevalence of *TP53* mutations/*MDM2* amplifications in pediatric RMS (1.33% and 0%, respectively). The possible inactivation of p53 function by other mechanisms thus remains to be elucidated.

## 1. Introduction

Rhabdomyosarcoma (RMS) is the most common type of soft tissue sarcoma diagnosed in children under the age of 15 years contributing to approximately 4% of all childhood malignancies [1]. Two major subtypes, embryonal (ERMS) and alveolar rhabdomyosarcoma (ARMS), together comprise 80% of all rhabdomyosarcoma [2]. The predominant subtype is ERMS which is characterized by earlier age of onset and better survival compared to ARMS (70% versus 50%, resp.) [2, 3]. While ERMS is characterized by frequent loss of imprinting on chromosome 11p15, a region containing a number of imprinted genes, including *IGF2*, 80% of ARMS present with translocations, most frequently involving *PAX 3* or *7* and *FOXO* gene rearrangements [4, 5]. Young age of onset, a number of identified predisposing syndromes, and paucity of environmental and lifestyle risk factors all contribute to the widely accepted view that genetic aberrations may play an important role in RMS development [3, 5]. However, the etiology of RMS remains largely unknown primarily due to its rarity and diagnostic diversity [2].

*TP53* is the most commonly mutated gene in human cancer; however, the prevalence of *TP53* mutations varies greatly by cancer type [6]. This tumor suppressor gene is activated in response to DNA damage and mediates cell cycle arrest or induction of apoptosis. MDM2 is a key negative regulator of p53 expression; binding of MDM2 to p53 targets p53 for proteasomal degradation. Therefore, overexpression of MDM2 attenuates p53 function.

Missense mutations are the most common mechanisms of *TP53* inactivation [7]. Frequently mutations are clustered in exons 5 through 8 [7], the region coding for the DNA binding domain, where “hotspot mutations” have been described. In addition, a number of variants in the *TP53* gene have been described, and some of these, including SNP72 (rs no. 1042522, C/G), may modify the risk of cancer development [8]. SNP72 is a coding SNP producing proteins with slightly different properties. In particular, compared to the 72Arg (C allele) variant, the 72Pro (G allele) has a lower affinity for MDM2 [9]. This variant has been shown to affect the age of tumor onset in patients who carry a germline mutation in *TP53* [10]. Similarly, accelerated tumor formation in germline *TP53* carriers was observed in carriers of the minor allele (G) for the *MDM2* SNP309 [11, 12]. The *TP53* SNP72 C allele has been shown to interact with the *MDM2 *SNP309 G allele to amplify the risk of tumor onset at an earlier age in carriers [10].

The risk of RMS development increased in *TP53 *mutation carriers, but the role of the *TP53 *polymorphisms described above on the age of cancer onset has not been explored in sporadic RMS.

*TP53* mutations have been studied in RMS, but the majority of these studies had small sample sizes and the prevalence of reported mutations varied greatly (Table 1) [13–24]. Larger studies have also reported inconsistent data. For example, Mulligan et al. [18] and Taylor et al. [22] detected very low frequencies of *TP53* mutations, 2/31 and 1/20, respectively, while Takahashi et al. [21] reported a much higher mutation frequency (9/45). In addition, Taylor et al. [22] examined amplifications of the *MDM2* gene, a frequent mechanism for MDM2 overexpression in other tumor types, and detected a very low frequency of amplifications (2/20).

We have analyzed *TP53* mutations in the largest series of RMS tumors thus far, enabling the analyses by RMS subtypes, as well as MDM2 amplification status. In addition, we have examined whether *TP53* and *MDM2* polymorphisms may affect the age of RMS onset and have also explored the associations between these polymorphisms and specific tumor subtype and primary tumor site.

## 2. Material and Methods

### 2.1. Tumor Samples

Deidentified frozen RMS tumor tissues (*N* = 40) were obtained from the Cooperative Human Tissue Network (CHTN). Histopathology slides (*N* = 40) were obtained separately from CHTN and kindly provided for this study by Dr Brenda Weigel (University of Minnesota). Five of the histology slides corresponded to five of the frozen tumor tissues, and therefore the final sample collection included 75 different RMS patient tumors. CHTN houses the tissues of Children's Oncology Group's (COG) pediatric solid tumor bank, obtained after routine surgical resections performed at COG-affiliated institutions across the United States and Canada. Tumors were preserved either by snap-freezing in liquid nitrogen and storing at −80°C or by fixation in formalin, followed by paraffin embedding for histopathology slides. Central review of histologic slides and clinicopathologic data was performed by an anatomic pathologist at CHTN for each sample, and histologic subtypes (embryonal or ERMS; alveolar or ARMS) were determined. For a handful of samples, information on percentage of area of tissue involved by tumor and percentage of necrotic tumor cells was available. Review of all histopathology slides was performed by Carlos Manivel, M.D., at the University of Minnesota to estimate the percentage of tumor and necrosis in the samples.

### 2.2. Sequencing of the *TP53* Gene

DNA was isolated from frozen tissues by using DNeasy blood and tissue mini kit, while DNA from histopathology slides was isolated using QIAamp DNA Micro kit, according to the manufacturer's protocol (Qiagen, Valencia, CA). DNA amplification and *TP53* sequencing were performed at the International Agency for Research on Cancer (IARC) common sequencing service using standard procedures. Primer sequences, product lengths, and PCR conditions are available at http://www-p53.iarc.fr/Download/TP53_DirectSequencing_IARC.pdf. PCR products were purified using the enzyme ExoSap IT (USB), and 7 *μ*L of each purified product was sequenced by using BigDye Terminator v1.1 Cycle Sequencing Kit (Applied Biosystems) according to manufacturer's protocol. Sequencing primers were the same as the ones used for amplification. Sequencing reaction products were purified by using 96-well Multiscreen filtration plates (G50-Pharmacia-Millipore) and analyzed by a 16-capillary automated sequencer (ABI PRISM 3100 Genetic Analyzer, Applied Biosystems).

### 2.3. Analysis of *MDM2* Amplifications

*MDM2* amplification was analyzed by using differential PCR. The analysis was carried out as previously described, with some modifications [25]. Briefly, each DNA sample isolated from 22 frozen tissue tissues was added to a PCR mix with primers for *MDM2* and primers for dopamine receptor 2 (*DRD2*) reference sequence. The sequences of the primers were 5′-GAGGGCTTTGATGTTCCTGA-3′ (forward) and 5′-GCTACTAGAAGTTGATGG C-3′ (reverse) for *MDM2*, and 5′-CCACTGAATCTGTCCTGGTATG-3′ (forward) and 5′-GTGTGG CATAGTAGTTGTAGT GG-3′ (reverse) for *DRD2*. PCR products were analyzed by electrophoresis on 3.0% agarose gel and stained with ethidium bromide. The intensities of the DNA products were quantified by densitometry using ImageQuant Version 5.0 software (Molecular Dynamics, Inc., Sunnyvale, CA). The *MDM2*/*DRD2* ratio from normal blood DNA was 0.9, with a standard variation of 0.23. A value of more than 2.5 (2x mean + 3x SD) for the *MDM2/DRD2* ratio was regarded as positive for *MDM2* amplification [25].

### 2.4. Analysis of *MDM2* SNP309

DNA samples were genotyped for the single nucleotide polymorphism (SNP) *MDM2* 309 using the 5′ nuclease Taqman allelic discrimination assay including Taqman Universal PCR Master Mix (Applied Biosystems Inc., ABI, Foster City, CA), as previously described [11]. For each assay, there were four no template controls, four controls specific for the wild-type allele, and four controls specific for the variant allele. Completed PCR plates were analyzed using an ABI Prism 7900 Sequence Detection System (ABI) and Sequence Detection Software version 2.1 (ABI). Sample analyses were performed in duplicate.

### 2.5. Statistical Methods

Patient tumor characteristics and SNP classifications were compared using Chi-squared and Fisher's exact tests as appropriate for categorical data and *t*-tests and analysis of variance (ANOVA) for continuous variables. Means ± standard deviations (SD) are reported unless otherwise noted. A *P*-value of <0.05 was considered statistically significant. All analyses were performed using SAS version 9.2.

## 3. Results

### 3.1. Characterization of Patient Tumors

Clinicopathologic data included histologic subtype, primary site, age at diagnosis, gender, and race/ethnicity (Table 2). The majority of patients were non-Hispanic Caucasians (74.4%), while 6.7% of patients were Hispanic, and 9.3% were African Americans. There were 45 male and 30 female pediatric RMS patients ranging in age from 3 months to 18 years. There was no difference in mean age by histologic subtype: age for ERMS was 7.1 ± 5.1 years and for ARMS was 7.0 ± 5.1 years. A large diversity of primary sites showed that the tumors could arise almost anywhere in the body. When the sites were grouped, extremities represented the single most common site (*N* = 19; 25.3%), followed by genitourinary (*N* = 18; 24.0%), head and neck (*N* = 11; 14.7%), and abdomen (*N* = 9; 13.3%) (Table 2). Tumors occurring in extremities had predominantly ARMS histology (15/19), while genitourinary tumors had predominantly ERMS histology (15/18). Tumors in trunk and visceral locations all had ARMS histology. The age at diagnosis differed significantly with primary site (*P* = 0.035). The youngest age at diagnosis was observed for pelvic tumors (mean age ± standard deviation was 2.8 ± 1.0 years, resp.) and the oldest for genitourinary tumors (10.2 ± 5.0 years). Patients with abdominal tumors were significantly younger at diagnosis (*P* = 0.05). We also observed a significant difference (*P* = 0.012) between tumor primary sites by gender, with genitourinary and abdominal tumors predominantly observed in males (15/18 and 8/10, resp.) and head and neck tumors in females (9/11).

Seventy five RMS tumor samples were analyzed for *TP53* mutations in exons 4–9 by direct sequencing. We identified only a single *TP53* mutation present in exon 7, c.775G/T (p.D259Y). This missense mutation leads to loss of p53 function. The patient harboring this mutation was a one-year-old Caucasian boy with ERMS tumor on the right thigh, tumor stage was not obtained. By analyzing sequencing data, we have also observed a number of variants in the *TP53* gene. In addition to SNP72, the most prevalent SNP was rs no.1800372 (A/G at third base of codon 213, not changing protein sequence), observed in 4 RMS tissues (5.3%). The prevalence of G allele in our study was slightly higher than the one reported in dbSNP for the US population (1%).

*MDM2* amplification frequency was analyzed in 22 RMS tumor samples by differential PCR. No *MDM2* amplification was detected in DNA isolated from any of these samples (data not shown).

We have also analyzed the polymorphisms in *MDM2* SNP309 and *TP53* SNP72 in DNA isolated from frozen-tumor samples (*N* = 40) (Table 3). For *MDM2* SNP309 we observed the following genotype frequencies: 40% for T/T, 40% for T/G, and 20% for G/G. These frequencies are similar to those previously reported in healthy Caucasians [11, 26, 27]. The mean age of diagnosis was 6.4 years for T/T, 6.7 years for T/G, and 7.6 years for G/G *MDM2* SNP alleles and are not statistically different from each other (*P* = 0.85). For the *TP53* SNP72, the frequencies of C/C, C/G, and G/G genotypes in rhabdomyosarcoma were 60%, 30%, and 10%, respectively, which were similar to those in healthy Caucasians [28, 29]. Although the mean age of tumor onset in C/C genotype carriers was slightly lower compared to those with C/G or G/G genotypes (6.3 ± 5.2 versus 7.5 ± 4.8, and 7.0 ± 4.8, resp.), these differences were not significant (*P* = 0.78). We have also analyzed the effect of either polymorphism (*MDM2* SNP309 or *TP53* SNP72) on the tumor subtype and observed no association. Likewise, there was no association between any SNP and tumor primary site.

Because *MDM2* SNP309G and *TP53* SNP72Arg have been reported to act synergistically and were together associated with the lowest age of cancer onset in carriers [10], we examined whether these variants may act together in sporadic RMS to confer increased risk of cancer onset at a younger age (Table 4). Only a single patient possessed the protective genotype, reported to be associated with later cancer onset in germline *TP53* mutation carriers [10] (0 at risk loci shown in Table 4), and although patients with less favorable genotypes appeared to be slightly younger at cancer diagnosis, these differences were not statistically significant (*P* = 0.78).

## 4. Discussion

A wide range of prevalences of *TP53* mutations in RMS were reported in the literature, with larger studies tending to report lower mutation frequencies. To address these inconsistencies, we have analyzed *TP53* mutations in exons 4–9 in 75 pediatric RMS tumor samples, as the majority of *TP53* mutations occur in exons 5–8. We detected only a single mutation in exon 7 (c.775G/T, p.D259Y), associated with loss of p53 activity [30]. This occurred in a 1-year-old patient with ERMS. ERMS are characterized by an earlier age of onset compared to ARMS, with approximately half of all cases diagnosed before the age of 5 years. Interestingly, the tumor of this patient was located in the lower extremity, a site that is more frequently associated with ARMS tumors.

All tumor samples in this study came from the CHTN, which includes a central pathology review. However, the majority of samples did not have accompanying data on the percentage of tumor present in each sample or the prevalence of necrosis. We therefore performed hematoxilin staining of the available histopathology slides and reviewed all histopathology slides to complete these data. This helped us address the possibility that the observed low prevalence of mutations in these tumors may be due to a large content of normal tissue compared to tumor tissue in our samples, which might undermine the detection of mutations that may be present in tumor. However the pathology review determined that, except for two samples, that had approximately 60% of the sample composed of tumor tissue, at least 80% of all other samples was tumor tissue. All reviewed samples had over 90% viable cells present. Therefore, it is unlikely that the composition and integrity of our samples may have affected the observed low prevalence of TP53 mutations.

Differences in the methods used to identify mutations in earlier compared to more recent studies may partially account for the observed differences in mutation frequencies. All earlier studies used single strand conformation polymorphism (SSCP), a method shown to more frequently detect *TP53* mutation detection, compared to sequencing, which is current gold standard [31, 32].

Some clinicopathologic characteristics of our samples were in line with well-established differences between the two major RMS subtypes, including differences in primary sites, and equal male to female ratio among ARMS cases [2]. In contrast, earlier age of onset of ERMS compared to ARMS was not reflected in our samples (Table 2). Despite slightly older age of ERMS cases than anticipated, the proportion of very young children (up to 5 years of age) was substantial (34/75), indicating that the age may not explain the observed low mutation prevalence. Namely, among germline *TP53* carriers, RMS occured very early in life and many developed RMS before the age of 5 years [33]. By analogy, if sporadic RMS tumors harboring *TP53* mutations were more likely to occur in younger children, a study not including this age category would likely report lower prevalence of *TP53* mutations. There was a disproportionately higher number of males than females with ERMS in our study compared to the reported 1.3 male to female ratio. As we observed no gender differences, gender distribution was unlikely to affect our results.

To explore whether increased levels of MDM2 may provide an alternative mechanism to *TP53* gene mutation, we have analyzed *MDM2* gene amplifications. Overexpression or amplification has been observed in a number of human cancers [34, 35] suggesting that it may act as an alternative mechanism to attenuate wild-type p53 function. Although *MDM2* amplifications were frequently observed in soft tissue sarcoma [34, 35], the only study examining this in pediatric RMS has shown very low prevalence of *MDM2* amplifications (2 out of 20 samples) [22]. In addition, a study that included both pediatric and adult RMS patients showed a similar prevalence (3 out of 18 patients) [21]. Our study confirms the finding that the prevalence of *MDM2* amplifications in pediatric RMS may be low, as we detected no *MDM2* amplifications in the 22 samples we analyzed. In contrast to the previous suggestion that *MDM2* amplifications may be more likely found in ARMS tumors compared to ERMS [21, 36], we observed no amplifications in either tumor subtype.

Among the variants that affect p53 degradation, *MDM2* SNP309 was associated with earlier age of cancer onset among germline *TP53* mutation carriers [10]. In addition, this SNP was reported to be associated with younger age of onset of sporadic soft tissue sarcoma [11], but this study included insufficient number of RMS samples to determine whether this association would be also observed for RMS. In contrast, our study included a much larger number of pediatric RMS samples, and we found no association between *MDM2* SNP309 and younger age of tumor onset. Likewise, *TP53* SNP72 was reported to be associated with age of tumor onset in carriers, where it was also shown to interact with *MDM2* SNP 309 to further reduce the age of tumor onset. Such interactions have also been described in several sporadic human malignancies; therefore, we examined whether *TP53* SNP72 may act as a modifier in RMS. We found no effect of *TP53* SNP72 on the age of tumor diagnosis, nor any evidence of interaction between *TP53* SNP72 and *MDM2* SNP309 on the age of tumor onset.

Although our study was relatively large, it was focused on pediatric RMS. Therefore, the range of ages at tumor onset was narrower compared to other studies that reported modifying effects of the variants affecting p53 degradation in either germline *TP53* carriers or other types of sporadic cancer. In addition, molecular characteristics of RMS tumors that occur in children may differ from those that occur in adults. Therefore, it remains possible that any modifying effects of the variants analyzed here would have been observed if adult cases had been included in our study. Further studies involving both pediatric and adult RMS patients would be needed to explore this.

In conclusion, we observed low prevalence of *TP53* mutations and no *MDM2* amplifications in pediatric RMS. This is different from what has been found before, and due to the sample size, ours is likely a more accurate finding. Variants *TP53* SNP72 and *MDM2* SNP309 did not accelerate tumor development. *TP53* may therefore not play an important role in pediatric RMS development. The possible inactivation of p53 function by other mechanisms thus remains to be elucidated.

## Figures and Tables

**Table 1:** Frequency of **TP53 ** point mutations reported in rhabdomyosarcoma.

Study size	Authors	Exons sequenced	Number of mutations	Location of mutations	
				Exon 5–8	Other
	Stratton et al., [20]	5–8	0/4	—	
	Felix et al., [15]	4–8	1/6	Exon 6: R213P	
	Toguchida et al., [23]	2–11	0/4	—	
≤10	Latres et al., [17]	2–9	0/2		Exon 11: D393N
	Castresana et al., [13]	5–8	1/1	Exon 6: V218Y Y220C	
	Würl et al., [24]	4–9	2/6	Exon 7: G245S	
	Kusafuka et al., [16]	5–8	1/10	Exon 8: R273H	
	Nawa et al., [19]	5–8	0/2	—	
	Das et al., [14]	2–11	1/4		

>10	Mulligan et al., [18]	5–8	2/31	Exon 5: G137V Exon 8: R282W	
	Taylor et al., [22]	5−9	1/20	Exon 5: del nt 1004–1017	
	Takahashi et al., [21]	5−9	9/45	Exon 6: E204G, R209T, P223R Exon 7: M243T, G245C, N247D, R249G Exon 8: C291G, P295H	

Total TP53 mutations		18/135 (13.3%)			

**Table 2:** Patient and tumor characteristics.

Characteristics	Histologic subtype	
	ARMS (*N* = 37)	ERMS (*N* = 38)
Sex		
Male	18	27
Female	19	11

Mean age (STDEV), years		
Boys	5.5 (4.6)	7.4 (5.0)
Girls	8.5 (5.3)	6.0 (5.1)

Race/ethnicity		
Caucasian	26	30
African American	3	4
Hispanic	2	3
Other	6	1

Tumor site		
Head and neck	7	4
Upper extremity	5	1
Lower extremity	10	3
Trunk	3	0
Abdomen	2	8
Visceral	4	0
Retroperitoneal	2	4
Pelvic	1	3
Genitourinary	3	15

**Table 3:** Mean age of first tumor onset according to MDM2 SNP309 or p53 codon 72 genotype.

	Allele (frequency)	Genotype	Number of patients (%)	Mean age ± STDEV (years)
MDM2 SNP309	T (60%)	T/T	16 (40)	6.4 ± 5.2
		T/G	16 (40)	6.7 ± 5.4
	G (40%)	G/G	8 (20)	7.6 ± 3.7
		T/G + G/G	24 (60)	7.0 ± 4.8

P53 codon 72 SNP	Arg (75%)	Arg/Arg	24 (60)	6.3 ± 5.2
		Arg/Pro	12 (30)	7.5 ± 4.8
	Pro (25%)	Pro/Pro	4 (10)	7.0 ± 4.8
		Arg/Arg + Arg/Pro	36 (90)	6.7 ± 5.0

**Table 4:** Mean age of first tumor onset according to the combined MDM2 SNP309 or p53 codon 72 genotype.

Number of at risk loci	Corresponding genotypes	Number of patients	Mean age ± STDEV (years)
0	T/T + Pro/Pro	1	9.0 ± 0.0
1	(T/G or G/G + Pro/Pro) or (T/T + Arg/Arg or Arg/Pro)	18	6.2 ± 5.2
2	T/G or G/G + Arg/Arg or Arg/Pro	21	7.1 ± 4.8
